# A Comparison of Doppler Flow Parameters in the Ophthalmic Artery and Central Retinal Artery in Patients With Graves' Disease and Toxic Nodular Goiter

**DOI:** 10.3389/fendo.2019.00707

**Published:** 2019-10-17

**Authors:** Dorota Walasik-Szemplińska, Grzegorz Kamiński, Małgorzata Mańczak, Joanna Widłak, Iwona Sudoł-Szopińska

**Affiliations:** ^1^Ophthalmic Hospital “Sensor Cliniq”, Warsaw, Poland; ^2^Department of Endocrinology and Radioisotope Therapy, Military Institute of Medicine, Warsaw, Poland; ^3^Department of Gerontology, Public Health and Didactics National Institute of Geriatrics, Rheumatology and Rehabilitation, Warsaw, Poland; ^4^Division of Thyrology and Radionuclide Therapy, Bielanski Hospital, Warsaw, Poland; ^5^Department of Radiology, National Institute of Geriatrics Rheumatology and Rehabilitation, Warsaw, Poland; ^6^Department of Medical Imaging, Second Faculty of Medicine, Medical University of Warsaw, Warsaw, Poland

**Keywords:** Graves' disease, Graves' ophthalmopathy, nodular goiter, hyperthyroidism, color doppler ultrasonography, OA, CRA

## Abstract

**Purpose:** Despite unquestionable clinical usefulness of Clinical Activity Score, the evaluating system needs frequent supplementation. One of such diagnostic tools is Doppler imaging that is used for the analysis of flow in the retrobulbar vessels. The improvement of the reliability and sensibility of measurements could make Doppler imaging an everyday clinical tool and improve the efficacy of treatment in patients with active thyroid-associated orbitopathy. However, the systemic influence of hyperthyroidism on the orbital vessels can falsify the assessment of local inflammation severity.

**Methods:** To eliminate the influence of systemic hyperthyroidism on orbital vessels, we compared peak systolic velocity (PSV), end-diastolic velocity (EDV), and resistance index (RI) in the central retinal artery (CRA), and ophthalmic artery (OA) in patients with hyperthyroidism in the course of Graves' disease without any detectable orbital changes, (CAS = 0) and toxic nodular goiter.

**Results:** There were no statistically significant differences between the patients with Graves' disease and toxic nodular goiter in terms of the examined parameters in either of the arteries. However, higher PSV and RI and lower EDV in the CRA as well as higher PSV and EDV and unchanged RI in the OA were found in the patients with Graves' diseases and toxic nodular goiter.

**Conclusion:** Hyperthyroidism and hyperthyroidism-induced hyperkinetic flow have a systemic influence on the orbital vessels, irrespective of the cause of hyperthyreosis. Thus, it is necessary to compare the flow parameters in retrobulbar vessels in Graves' patients with the toxic nodular goiter patients to eliminate the systemic influence of hyperthyroidism on the orbital vessels.

## Introduction

One of the methods for the evaluation of orbital inflammation related with Graves' disease is the analysis of flow in retrobulbar vessels using Doppler imaging. Compared with healthy individuals, flow parameters in patients with thyroid-associated orbitopathy show statistically significant differences (1–5). A vessel that is the most susceptible to orbital morphological changes is the superior ophthalmic vein, where one can notice decreased flow velocity, stasis, or retrograde flow irrespective of the phase of the disease (infiltration or fibrosis). Some authors suggest that venous stasis is the cause rather than the effect of some ocular signs and symptoms, such as conjunctival chemosis, enlarged extraocular muscles, increased orbital fat volume, and exophthalmos. Moreover, a correlation has been observed between stasis in the superior ophthalmic vein and the occurrence of optic neuropathy (3, 4).

Although changes in orbital veins are relatively well-studied and documented, investigations addressing flow in orbital arteries are still rare. In available literature, authors compare flow parameters in orbital arteries in patients with thyroid-associated orbitopathy with those in healthy volunteers (1, 2, 5). The discrepancies between the measurements may result from differences in the classification criteria of patients with thyroid-associated orbitopathy based on the clinical activity score (CAS), developed by Mourits et al. and from the adopted study methods (6). The CAS, despite its unquestionable usefulness in the clinical practice, has numerous limitations (7). Supplementing the CAS with a retrobulbar flow analysis with Doppler imaging could translate into improved diagnosis and treatment efficacy, both regarding treatment initiation and type of therapy.

However, a condition necessary to enable the application of Doppler imaging in the assessment of thyroid-associated orbitopathy is increasing its specificity and acquiring reproducible and reliable results.

The alterations of the flow parameters described so far in patients with Graves' ophthalmopathy are at least partially associated with a systemic influence of excessive serum thyroid hormone levels. Both orbital veins and arteries are influenced by thyroid hormones, as are other vessels in the body. Studies addressing the influence of excessive thyroid hormone levels on vascular walls clearly indicate permanent arterial remodeling that takes place even upon normalization of thyroid-stimulating hormone (TSH) levels in response to treatment (8).

The hemodynamic consequences of elevated serum thyroid hormone levels, irrespective of their cause, directly result in increased heart rate, both while resting and upon exertion, increased blood volume in the vascular bed, increased ejection fraction, increased myocardial contractibility, and increased stroke volume, all of which induce peripheral vascular hyperkinetic flow (9, 10).

Hence, it seems significant to study the flow parameters in the orbital vessels in patients with hyperthyroidism of various etiologies. The comparison of peak systolic velocity (PSV), end-diastolic velocity (EDV), and resistance index (RI) in the orbital arteries in patients with Graves' disease and toxic nodular goiter may eliminate the systemic effect of hyperthyroidism and reveal changes specific for Graves' disease.

The aim of the study was to compare the flow parameters in the CRA and OA in patients with hyperthyroidism in the course of Graves' disease without any detectable orbital changes (CAS = 0), and toxic nodular goiter and in healthy individuals.

## Materials and Methods

This prospective study was conducted from October 2012 to April 2016 in the Department of Ophthalmology and Department of Medical Imaging in the Masovian Bródno Hospital.

### Group Characteristics

**Figure 1:**
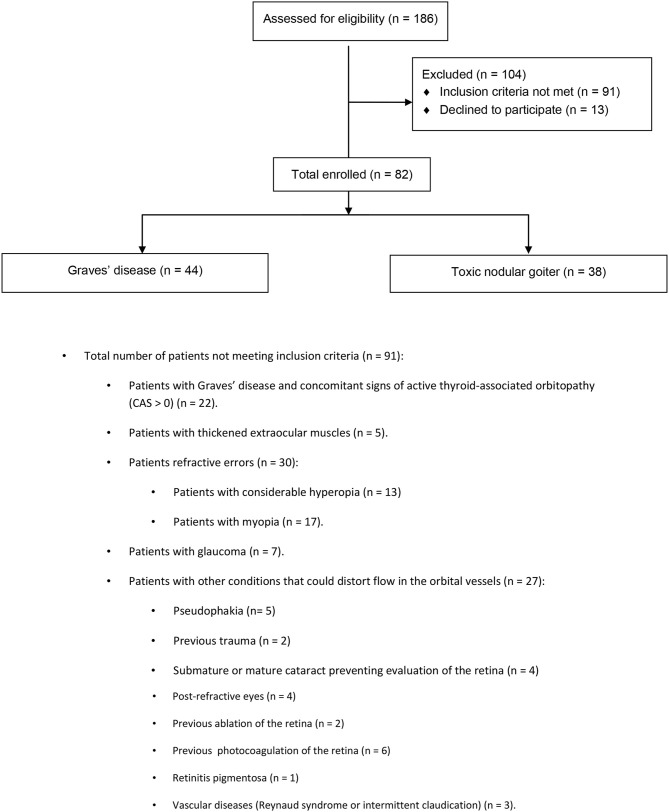
CONSORT study flow chart.

Eighty-two hyperthyroid patients were enrolled: 44 patients with Graves' disease and 38 patients with toxic nodular goiter. Some patients were excluded from the study, such as those with Graves' disease and with concomitant signs of active thyroid-associated orbitopathy (CAS > 0), thickened extraocular muscles, refractive errors, considerable hyperopia, myopia, glaucoma, and other conditions that could distort flow in the orbital vessels. For further details, refer to the CONSORT study flow chart (Figure 1).

Eligible patients were recruited from the Endocrinology Outpatient Clinic. Diagnosis of both Graves' disease and toxic nodular goiter was made by an endocrinologist based on clinical symptoms and biochemical tests [TSH, free thyroxine (FT4), free triiodothyronine (FT3), TSH receptor autoantibodies (TRAb), and anti-thyroid peroxidase antibodies (ATPO)], and was consistent with the published criteria (11, 12). All included patients underwent thyroid ultrasound and thyroid scintigraphy. From the time of diagnosis, patients had been managed by GPs or endocrinologists outside the clinical center until treatment ineffectiveness or adverse effects prompted referral to the Isotope Endocrine Clinic for assessment for eligibility for radioiodine therapy.

The group of patients with Graves' disease consisted of 31 females (70%) and 13 males (30%); whereas, the group of patients with toxic nodular goiter included 35 females (92%) and 3 males (8%). The control group included 45 healthy volunteers; 29 females (64%) and 16 males (36%). All study participants (both patients and controls) were Caucasian. All patients expressed written consent to participate in accordance with the Declaration of Helsinki. The study was approved by the Bioethic Comitee of Medical University of Warsaw (no. AKBE/201/17).

The mean age of the participants was 46 years in the Graves' disease group, 59.5 years in the group with toxic nodular goiter, and 63 years in the control group. The groups were homogeneous in terms of the duration of the disease; the mean duration of the disease was 5 years (IQR: 2–8 years) for Graves' disease and 3 years (IQR: 2–7 years) for toxic nodular goiter (*p* = 0.342).

All patients were under permanent care of an endocrinology clinic and were treated with oral thyreostatic drugs, being either euthyroid or subclinically hyperthyroid despite treatment in both groups. In the Graves' disease group, there were 17 euthyroid and 27 subclinically hyperthyroid patients, and in the goiter group there were 17 and 21, respectively. All eligible patients were treated by an endocrinologist at the study site for at least 0.5 years and had available medical history. Before enrolment, none of the patients had been treated with radioactive iodine or strumectomy. All patients were treated with oral antithyroid drugs [propylthiouracil (Thyrosan) or thiamazole (Metizol)]. Neither the type of an active substance nor the dose was the inclusion or exclusion criterion. In the Graves' disease group, treatment was carried out intermittently (due to periodic remissions), as noted in the medical history. Five patients with Graves' disease had previously discontinued treatment as a result of non-compliance with the recommendations of the attending physician (an endocrinologist). Because these patients restarted treatment more than 0.5 years before entering the study, they were eligible to participate.

The groups were also compared in terms of other factors, such as smoking, eye displacement (Hertel exophthalmometry), and intraocular pressure.

### Examinations Performed

#### Biochemical Tests

All patients had measured serum TSH, FT3, and FT4 levels. Additionally, Graves' disease patients underwent tests for TRAb, and patients with toxic nodular goiter were studied for ATPO. The cut-off point was 2.0 IU/L for TRAb (normal range: 0–2 IU/L) and 50 IU/mL for ATPO (normal range: 0–50 IU/mL), in accordance with referential ranges of a given laboratory. All assays were performed with the LIAISON® XL chemiluminescence analyzer.

#### Ophthalmologic Examination

All patients underwent a complete ophthalmic examination, including far and near vision acuity tests with autorefractometry, air-puff, and applanation intraocular pressure tests, pupillary reflex tests (direct, indirect, RAPD), pseudochromatic Ishihara tests for color perception, eyeball displacement assessment using Hertel exophthalmometer, and eyeball motility tests. The anterior segment and the fundus of the eye were assessed biomicroscopically using a 78D Volk lens. The ophthalmic examination followed the guidelines of the European Group on Graves' Orbitopathy. Moreover, patients with Graves' disease underwent the assessment of thyroid-associated orbitopathy by using the CAS.

#### Blood Pressure and Heart Rate Measurements

The blood pressure and heart rate of all study participants were measured. The measurements were taken not later than 30 min after retrobulbar flow assessment.

#### Ultrasound Examination

##### Thyroid ultrasound

The following parameters were studied in all study patients: size (three dimensions) and volume; echogenicity; internal structure; borders; presence of calcifications; and blood supply (vascularization) of the entire parenchyma and focal lesions (color Doppler or power Doppler). In 17 patients, thyroid ultrasound provided indications for fine-needle aspiration based on the European Thyroid Association Guidelines (13). None of the patients were found to have malignancies.

##### Orbital ultrasound

Inclusion criteria required that all patients underwent orbital ultrasound once. Thickness and echogenicity of the ocular muscles were assessed. B-mode ultrasound examination of the eyeballs and orbits was performed using the Aviso platform (Quantel Medical) and a linear probe with a frequency of 10 MHz. The evaluation assessed the thickness of the extraocular muscles: the superior rectus, inferior rectus, lateral rectus, and medial rectus. The obtained values were compared between both eyes and with the population norm (14). The patient was excluded from the study if any of the following conditions was detected: thickening of any of the ocular muscles relative to the population norm; asymmetry between the muscles in the right and left eye; or changes in muscle echogenicity potentially indicative of edema. Hypoechogenicity and consistency of the orbital fat was also assessed qualitatively. If a decrease in echogenicity, a change in consistency or empty spaces in the orbital fat were detected, the patient was excluded from the study.

After study inclusion, Doppler ultrasound was used to evaluate the extraocular vessels (i.e., the CRA and OA) in both eyes. Flow in the central retinal arteries and ophthalmic arteries in both eyes was evaluated with the Doppler method on the day of the ophthalmic examination. The scans were conducted using ACUSON X300 PE (Siemens AG) with a linear probe VF13-5 of the frequency range 4.4–13 MHz. The scans were performed in the supine position after a 15 min rest. The contact method was used through closed eyelids with eyeball position at 0°. The probe was applied perpendicularly to the eye surface through acoustic gel with no pressure exerted on the eyeball. Doppler signal enhancement was set below the artifact threshold, thus acquiring good color saturation. The pulse repetition frequency (PRF) was in each case adjusted to flow velocity in a given vessel. When the vessel was located, a square Doppler gate with a side of 2 mm was set. The maximum correction of the insonation angle was 30°.

**Figure 2:**
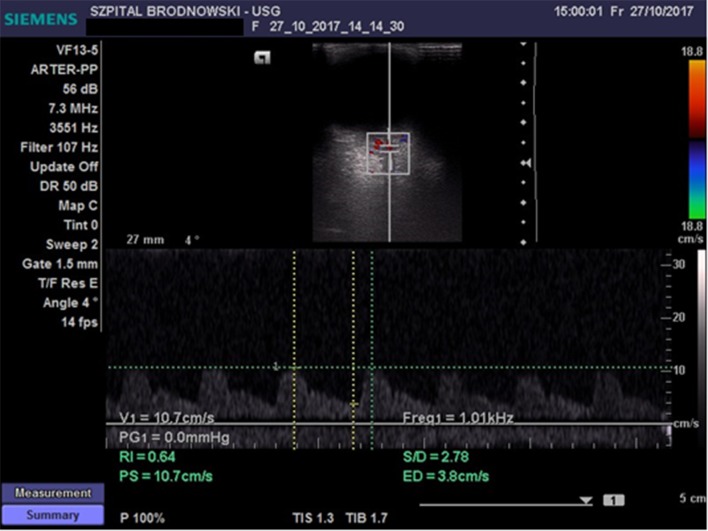
Flow velocity waveform in the central retinal artery.

**Figure 3:**
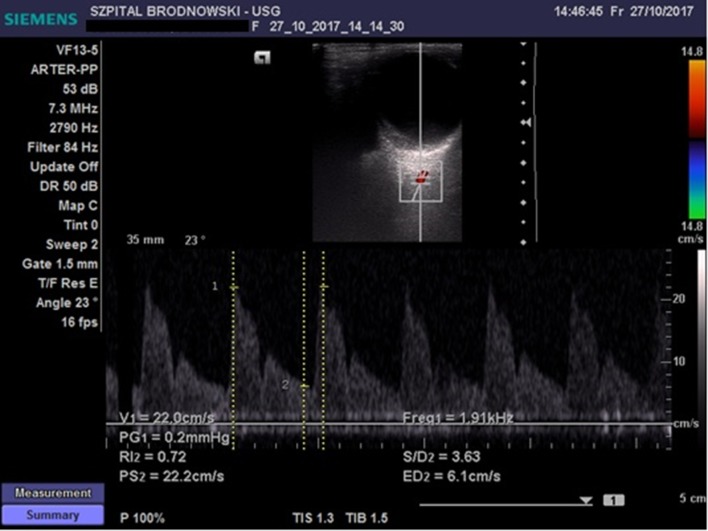
Flow velocity waveform in the ophthalmic artery.

The CRA was located in the region of the optic disc; flow velocity was recorded 2–3 mm below the posterior eyeball wall (Figure 2) Flow velocity in the OA was recorded at the depth of 35–36 mm (Figure 3). All ultrasound examinations of the retrobulbar arteries were conducted by a single person.

The following parameters were evaluated: peak (maximum) systolic velocity (PSV), EDV, and RI, defined as a quotient of the difference between maximal and minimal velocity divided by the maximal velocity. Three independent measurements of the said parameters were taken for each artery, and the resultant numerical values were averaged. Standard deviation for the resultant values of the flow parameters (PSV, EDV, and RI) was calculated.

In the healthy individuals, an additional examination of flow in the carotid arteries was conducted using Philips HD7 XE.

The flow parameters in the central retinal and ophthalmic arteries were correlated with TSH, FT3, and FT4 levels in both groups of hyperthyroid patients. The results were additionally correlated in patients with the serum TRAb level in Graves' disease and with the ATPO level in toxic nodular goiter.

### Statistical Calculations

The statistical calculations were performed in Statistical v. 10. The normality of distribution of continuous variables was tested with the Shapiro–Wilk test. The distribution of continuous variables analyzed in this study was significantly different from that of normal distribution. That is why median values and interquartile ranges (IQR) were used in their description. The nominal variables were presented in sizes and percentage values.

The Mann–Whitney *U*-test was used for the comparison of continuous variables between two groups, while the Kruskal–Wallis test was used to make comparisons between three groups. The nominal variables were compared using the χ2 test or χ2 test with Yates's correction (in the case of any expected size <5). Correlations between variables were tested by determining the Spearman's correlation coefficient. The statistical significance level was *p* < 0.05.

## Results

### Patient Group Comparison

No statistically significant differences were found between the patients with Graves' disease and those with toxic nodular goiter in terms of serum TSH, FT3, and FT4 levels; disease duration; smoking status; and intraocular pressure. Despite the absence of a statistically significant difference in the serum TSH concentration between the two groups, it must be emphasized that both groups included euthyroid and subclinically hyperthyroid patients in spite of the implemented therapy. Hence euthyroid patients (TSH: 0.35–4.0 uIU/mL) and hyperthyroid patients (TSH <0.35 uIU/mL) were selected from each group.

In each of the patient groups (Graves' disease and goiter), the subgroups of euthyroid and subclinically hyperthyroid patients were analyzed. Parameters of flows in CRA and OA were compared between patients in euthyreosis and in subclinical hyperthyreosis in both patient groups and between the groups. No statistically significant differences were found in PSV, EDV and RI in the CRA, and in EDV and RI in the OA between euthyroid and subclinically hyperthyroid patients in either patient group (Graves' disease and goiter). The difference in EDV in the OA between subclinically hyperthyroid and euthyroid patients in the goiter patient group was at the limit of statistical significance. Similarly, the difference in PSV in the OA between euthyroid patients with Graves' disease and euthyroid patients with goiter approached statistical significance.

Due to the lack of statistically significant differences in the flow parameters in the CRA and OA between patients in euthyreosis and those in subclinical hyperthyroidism, we considered it reasonable to proceed without dividing the groups according to TSH concentration.

The groups differed in terms of sex distribution. The toxic nodular goiter group included more women than men compared with the Graves' disease group. Women constituted 92% in the former group and 70% in the latter. In the control group, women accounted for 64% of the participants (*p* < 0.004) (Table 1).

**Table 1:** Group characteristics: systolic (SBP) and diastolic blood pressure (DBP), heart rate (HR), TSH, FT3, FT4, intraocular pressure (IOP), exophthalmometry (Hertel), sex distribution, disease duration, and smoking status.

	**Toxic goiter *N* = 38**	**Graves' disease *N* = 44**	**Controls *N* = 45**	***p* statistical significance level**
**General characteristics**				
Sex (female) (*n*, %)	35 (92%)	31 (70%)	29 (64%)	0.004
Age (years)	59.5 (50–67)	46 (34–57)	63 (44–73)	<0.001
Smoking (*n*, %)	11 (29%)	14 (32%)	9 (20%)	0.424
SBP (mm Hg)	135 (126–140)	140 (126–140)	131 (126–140)	0.651
DBP (mm Hg)	80 (78–87)	80 (78–87)	80 (78–87)	0.581
HR (bpm)	77 (78–87)	86 (78–87)	72 (78–87)	<0.001
**Thyroid**				
Disease duration (years)	5 (2–8)	3 (2–8)	–	0.342
TSH (uIU/mL)^a^	0.252 (0.041–1.505)	0.043 (0.006–1.554)	–^d^	0.059
FT3 (pg/mL)^b^	3.80 (2.9–5.7)	3.70 (2.85–4.54)	–^d^	0.423
FT4 (ng/dL)^c^	4.90 (1.16–10.86)	1.52 (1.11–7.32)	–^d^	0.165
TRAb^e^	–	17.65 (6.90–30.99)	–	–
ATPO^f^	44.50 (34.5–172.0)	–	–	–
**Eyes**				
Both eyes	*N* = 76	*N* = 88	*N* = 88	
IOP (mm Hg)	16 (14–18.5)	16 (14.5–19)	16.5 (14.5–18)	0.787
Hertel (mm)	16 (14–17)	18 (16–20)	16 (14.5–17)	<0.001

a *Normal range for TSH: 0.35–4.0 uIU/mL*.

b *Normal range for FT3: 1.8–4.2 pg/mL*.

c *Normal range for FT4: 0.8–1.9 ng/dL*.

d *No TSH, FT3 or FT4 measurements were performed in the control group, all of whom were healthy with no symptoms or positive family history for thyroid disease. GPs provided attestations confirming good health status for patients >60 years old*.

e *Normal range for TRAb: 0–2 IU/L*.

f *Normal range for ATPO: 0–50 IU/mL*.

Although the groups differed in terms of age, the mean age in the toxic goiter group was 59.5 years, whereas that in the Graves' disease group was 46 years. The mean age of the controls was 63 years (*p* < 0.001).

While comparing the studied groups in terms of the eyeball position in exophthalmometry using Hertel exophthalmometer, a statistically significant difference was observed between the values of patients of the Graves' disease compared with those of toxic nodular goiter and controls. The median eyeball displacement in the Graves' disease patients was 18 mm, whereas the value in the controls and patients with toxic nodular goiter was 16 mm (*p* < 0.001). However, it must be underlined that the values obtained in both groups fell within the population referential ranges (15).

In both patient groups and in the controls, there were no statistically significant differences in systolic and diastolic blood pressure. The patients with Graves' disease and toxic nodular goiter had higher heart-rate values than the controls (*p* < 0.001) (Table 1).

### Comparison of the Flow Parameters in the CRA and OA Between the Right and Left Eye

The flow parameters in the CRA and OA in the right and left eye were compared in both groups of hyperthyroid patients and in the control group. There were no statistically significant differences in PSV, EDV, and RI between the eyes in any of the groups. Moreover, intraocular pressure (IOP) and exophthalmometric values were compared between the right and left eye in all three groups. There were no statistically significant differences in the investigated parameters.

As there were no statistically significant differences in the studied parameters between the right and left eye in any of the groups, the further statistical analysis involved only one eye (left) in each patient from every group, as recommended in the statistical analysis methods (16, 17).

The flow parameters in the CRA and OA were compared between the Graves' disease and toxic nodular goiter patients. The flow parameters obtained in both groups were compared with the parameters obtained in the controls.

### Comparison of the Flow Parameters in the CRA

There were no statistically significant differences in the compared parameters, i.e., PSV, EDV, and RI, in the CRA between the patients with Graves' diseases and toxic nodular goiter (Table 2).

**Table 2:** Comparison of blood flow parameters (PSV, EDV, RI) in central retinal artery in Graves' disease and toxic nodular goiter patients.

**Parameter**	**Graves (left eye)**		**Goiter (left eye)**		***p* statistical significance level**
	***N***	**Median (IQR)**	***N***	**Median (IQR)**	
PSV CRA (cm/s)	44	12.64 (11.04–13.85)	38	12.75 (11.70–14.60)	0.850
EDV CRA (cm/s)	44	3.13 (2.70–3.80)	38	3.20 (2.50–3.87)	0.764
RI CRA	44	0.75 (0.71–0.79)	38	0.75 (0.73–0.79)	0.743

However, the comparisons of the flow parameters in the CRA between all hyperthyroid patients and controls revealed statistically significant differences in PSV, EDV, and RI; the hyperthyroid patients presented higher PSV than the controls (*p* = 0.001), whereas EDV was lower in the hyperthyroid patients compared with the healthy individuals (*p* = 0.008). Due to these flow velocity alterations, RI was higher in the hyperthyroid patients (*p* < 0.001) (Table 3).

**Table 3:** Comparison of blood flow parameters (PSV, EDV, RI) in central retinal artery in hyperthyroid patients and healthy controls.

**Parameter**	**Graves + Goiter (left eye)**		**Controls (left eye)**		***p* statistical significance level**
	***N***	**Median (IQR)**	***N***	**Median (IQR)**	
PSV CRA (cm/s)	82	12.66 (11.23–14.10)	45	11.53 (10.33–12.50)	0.001
EDV CRA (cm/s)	82	3.14 (2.63–3.80)	45	3.50 (3.16–3.93)	0.008
RI CRA	82	0.75 (0.72–0.79)	45	0.70 (0.66–0.73)	<0.001

Because of these differences between the hyperthyroid patients and healthy individuals, the flow parameters in the CRA were compared separately between the healthy individuals and both patient groups.

Peak systolic velocity and RI were statistically significantly higher, whereas EDV was lower in both groups of hyperthyroid patients compared with the controls (Table 4).

**Table 4:** Comparison of blood flow parameters (PSV, EDV, RI) in central retinal artery in Graves' disease, toxic nodular goiter, and healthy controls.

**Parameter**	**Median (IQR)**		**Median (IQR)**		***p* statistical significance level**
	***N***	**Goiter (left eye)**	***N***	**Controls (left eye)**	
PSV CRA (cm/s)	38	12.75 (11.70–14.60)	45	11.53 (10.33–12.50)	0.003
EDV CRA (cm/s)	38	3.20 (2.50–3.87)	45	3.50 (3.16–3.93)	0.033
RI CRA	38	0.75 (0.73–0.79)	45	0.70 (0.66–0.73)	<0.001
	***N***	**Graves (left eye)**	***N***	**Controls (left eye)**	
PSV CRA (cm/s)	44	12.64 (11.04–13.85)	45	11.53 (10.33–12.50)	0.007
EDV CRA (cm/s)	44	3.13 (2.70–3.80)	45	3.50 (3.16–3.93)	0.016
RI CRA	44	0.75 (0.71–0.79)	45	0.70 (0.66–0.73)	<0.001

### Comparison of the Flow Parameters in the OA

The PSV values in the OA of the patients with Graves' disease were not statistically significantly different from the PSV in the OA of the patients with toxic nodular goiter. Also, EDV and RI in the OA showed no statistically significant differences in either group (Table 5).

**Table 5:** Comparison of blood flow parameters (PSV, EDV, RI) in ophthalmic artery in Graves' disease and toxic nodular goiter patients.

**Parameter**	**Graves (left eye)**		**Goiter (left eye)**		***p* statistical significance level**
	***N***	**Median (IQR)**	***N***	**Median (IQR)**	
PSV OA (cm/s)	44	33.00 (29.41–38.28)	38	35.33 (29.06–40.22)	0.445
EDV OA (cm/s)	44	9.03 (6.15–10.60)	38	8.13 (7.16–10.40)	0.959
RI OA	44	0.73 (0.70–0.80)	38	0.74 (0.72–0.80)	0.333

However, the comparisons of the flow parameters in the OA between the patients with hyperthyroidism, irrespective of its cause, and the controls revealed statistically significant differences in PSV and EDV. The hyperthyroid patients presented higher PSV (*p* < 0.001) and EDV (*p* = 0.009) than the controls. Despite PSV and EDV alterations, RI showed no statistically significant differences in the patient groups (Table 6).

**Table 6:** Comparison of blood flow parameters (PSV, EDV, RI) in ophthalmic artery in hyperthyroid patients and healthy controls.

**Parameter**	**Graves + Goiter (left eye)**		**Controls (left eye)**		***p* statistical significance level**
	***N***	**Median (IQR)**	***N***	**Median (IQR)**	
PSV OA (cm/s)	82	34.02 (29.06–39.70)	45	28.06 (26.36–32.10)	<0.001
EDV OA (cm/s)	82	8.52 (6.43–10.57)	45	7.50 (4.93–9.26)	0.009
RI OA	82	0.74 (0.71–0.80)	45	0.75 (0.71–0.80)	0.720

A PSV increase, EDV decline, and stable RI were observed both in the patients of Graves' disease and toxic nodular goiter compared with the healthy controls (Table 7).

**Table 7:** Comparison of blood flow parameters (PSV, EDV, RI) in ophthalmic artery in Graves' disease, toxic nodular goiter, and healthy controls.

**Parameter**	**Median (IQR)**		**Median (IQR)**		***p* statistical significance level**
	***N***	**Goiter (left eye)**	***N***	**Controls (left eye)**	
PSV OA (cm/s)	38	35.33 (29.06–40.22)	45	28.06 (26.36–32.10)	<0.001
EDV OA (cm/s)	38	8.13 (7.16–10.40)	45	7.50 (4.93–9.26)	0.032
RI OA	38	0.74 (0.72–0.80)	45	0.75 (0.71–0.80)	0.938
	***N***	**Graves (left eye)**	***N***	**Controls (left eye)**	
PSV OA (cm/s)	44	33.00 (29.41–38.28)	45	28.06 (26.36–32.10)	<0.001
EDV OA (cm/s)	44	9.03 (6.15–10.60)	45	7.50 (4.93–9.26)	0.020
RI OA	44	0.73 (0.70–0.80)	45	0.75 (0.71–0.80)	0.516

Because of differences in sex distribution in all groups, the flow parameters in the CRA and OA were compared between women and men in every group (including the control group). There were no statistically significant differences. In the patients with toxic nodular goiter, analysis of the flow parameters was done in relation to the ATPO value. Flow parameters in the CRA and OA were not correlated with ATPO values. Moreover, there was no correlation between the flow parameters in the CRA and OA and serum TRAb value in the patients with Graves' disease. No correlation was found between either the systolic blood pressure or diastolic blood pressure and PSV, EDV, or RI values in either the CRA or OA. Similarly, no correlations were found between either patient age or disease duration and vessel flow parameters. A weak positive correlation was observed between age and PSV in the OA in patients with goiter, but this requires further evaluation in a larger group of patients. Similarly, no correlations were found between either patient age or disease duration and vessel flow parameters in the CRA or OA among patients with Graves' disease. In the control group, age was positively correlated with RI in both the CRA and OA, and negatively correlated with EDV in the OA. Due to the analysis of many variables, the level of statistical significance was reduced to *p* < 0.01. In addition, no correlations were found among PSV, EDV, RI and TSH, FT3, FT4 levels; and disease duration in both examined arteries.

## Discussion

A search for diagnostic tools that might improve classification of patients with thyroid-associated orbitopathy may have important clinical implications and may improve treatment outcomes. Exploring the pathophysiological mechanisms of orbital changes induced by the activation of specific antigens under the influence of elevated thyroid hormone levels is a key to enable the CAS with new criteria of transition from qualitative assessment to quantitative evaluation of the patients.

The current reports suggest that the analysis of flow parameters in the orbital arteries is correlated with the severity of inflammation in the course of thyroid-associated orbitopathy. It may be considered a reliable and non-invasive diagnostic tool (1–3).

In everyday clinical practice, a thorough assessment of disease activity is based on magnetic resonance imaging and computed tomography of the orbits and/or orbital soft-tissue biopsy. However, these methods are often unavailable, invasive (biopsy), expensive (MRI), and related with radiation exposure (CT).

Altered flow parameters in Doppler imaging may be an additional risk factor of exacerbation of thyroid-associated orbitopathy and may become the basis for early implementation of treatment or for taking a decision about early orbital decompression. Moreover, the cut-off point (3 or 4) for the diagnosis of active disease is usually difficult to determine, particularly when signs and symptoms are asymmetrical in both eyes. However, Doppler evaluation of orbital arteries can be used in daily practice only when its sensitivity is improved and a right reference for the obtained values is determined. Flow parameter alterations must be specific enough to enable the differentiation of orbital inflammation from hemodynamic changes occurring in all peripheral vessels.

The present study is a comparison of the flow parameters between patients with Graves' disease and hyperthyroidism due to toxic nodular goiter. The aim of the study was to evaluate the influence of hyperthyroidism and systemic hemodynamic changes on the orbital vessels.

The groups differed in terms of sex and age distribution. Women prevailed in all the groups. According to epidemiological data, hyperthyroidism occurs in approximately 0.5–2% of females of the Caucasian race and is 10 times more common in women than in males (18).

The differences in the mean age of the patients in the presented groups are also consistent with published epidemiological data. In regions without iodine deficiency, there are two hyperthyroidism incidence peaks: the first is observed between the age of 20 and 49 and is associated with Graves' disease, whereas the other in found at approximately 70 years of age and is related with toxic nodular goiter. Hence the differences in sex and age in the presented groups result from the specificity of these diseases and are consistent with population studies (18).

However, that our study had no epidemiological implications. That is why no conclusions about sex distribution, age, or incidence of hyperthyroidism in the studied population can be drawn. Neither age nor sex of patients was the inclusion or exclusion criterion.

Hertel exophthalmometry revealed a statistically significant difference in the eyeball position between the Graves' disease group and both the patients with toxic nodular goiter and controls.

Despite the lack of other signs of thyroid-associated orbitopathy, the eyeball position in the Graves' disease patients differed by 2 mm from that in other groups. However, the values obtained in all groups fell within the referential ranges for the Caucasian race (15).

Peak systolic velocity, EDV, and RI values in the CRA and OA were compared between the right and left eye in all three groups. There were no statistically significant differences between the investigated parameters in any of these groups. The consistent data obtained in all the groups indicate high reliability and reproducibility of the measurements and indirectly suggest that there were no signs of orbitopathy in the patients with Graves' disease. The lack of differences between the right and left eyes confirmed the need for a further statistical analysis of these parameters in one eye of each patient (16, 17).

The comparison of flow parameters in the CRA in the patients with Graves' disease without any signs of orbitopathy and in healthy controls confirmed earlier reports about statistically significant changes in PSV, EDV, and RI (3, 19). Our research demonstrates a significant increase in PSV (*p* = 0.007) and RI (*p* < 0.001) and a decrease in EDV (*p* = 0.01) in the patients compared with the controls.

Although, analogous changes occur in the CRA in patients with Graves' disease without any signs of orbitopathy and in patients with toxic nodular goiter, according to our knowledge, there have been no studies assessing flow parameters in the retrobulbar orbital vessels in patients with toxic nodular goiter. The present study shows no statistically significant differences in PSV, EDV, and RI between the patients with Graves' disease and those with toxic nodular goiter.

The results suggest a direct influence of enhanced cardiac output, cardiac contractility, and hemodynamic changes that are typically observed in hyperthyroid patients. Although the decreased EDV values in both groups of the hyperthyroid patients compared with the healthy individuals are intriguing, similar alterations have been reported by Plange in the posterior ciliary arteries in patients with normal-tension glaucoma (20). The cause of a decline of EDV values in slight arteries compared with healthy individuals remains unknown. Nonetheless, autoregulation disorders in response to NO and cGMP concentration changes in the slightest arteries may be responsible for flow parameter alterations (21). What is known for certain is that EDV is much more sensitive to hemodynamic disorders than PSV. This has been confirmed by Rosengarten and Satilmis who have demonstrated a negative correlation of glaucoma progression with EDV changes (22, 23).

In light of the latest studies, thyroid hormones have a potent vasodilatory effect by increasing NO, adrenomedullin, and adenosine production (24). In measurements of flow parameters, this should result in decreased vascular resistance in hyperthyroid patients. Hence, it is intriguing why all the published studies addressing flow in the retrobulbar vessels report increased RI in Graves' disease patients. The cause of an increase in vascular resistance in the CRA in Graves' disease patients is a statistically significant decrease in EDV, which results in increased RI as a mathematical function of PSV and EDV.

The observed EDV reduction in hyperthyroid patients may be associated with the overfilling of the CRA, which is a vessel with a small diameter. Increased volume of blood flowing into the vessel of a diameter of approximately 166 um results in decreased EDV and increased RI (25). Another probable mechanism of an increased vascular RI is a distorted autoregulation response to persistently increased volume of inflowing blood (24).

Poiseuille's—Hagen law shows the dependence between the vessel radius, flow velocity and the systemic resistance.

$$\begin{array}{l} \text{V}=\frac{\Delta \text{P}\pi {\text{xr}}^{4}}{8\text{I}\eta }\;\\ \frac{\Delta \text{P}}{\text{I}}-\text{pressure gradient}\\ \eta -\text{blood viscosity}\\ \text{r}-\text{vessel radius}, \end{array}$$

if we assume:

$$\begin{array}{l} \text{V}=\frac{\Delta P}{\text{R}} \end{array}$$

It follows:

$$\begin{array}{l} \text{R}=\frac{8\text{Iη}}{\pi {\text{r}}^{4}} \end{array}$$

It means that the resistance index is the higher the smaller radius of the vessel. In the conditions of hyperkinetic flow, the RI rises in the central retinal artery.

The analysis of the results obtained in the euthyroid patients and in the patients with clinically overt hyperthyroidism in both the Graves' disease group and the toxic nodular goiter group revealed that TSH is not correlated with PSV, EDV, and RI in the investigated vessels. Moreover, no statistically significant differences were observed between the flow parameters in the euthyroid patients (TSH: 0.35–4.0 uIU/mL) and hyperthyroid patients (TSH <0.35 uIU/mL). TSH concentration is not a factor that directly induces vascular hemodynamic changes. This thesis has been confirmed in earlier publications.

Kurioka et al. (19) reported increased RI values in the CRA in Graves' disease patients without any signs of orbitopathy compared with healthy individuals. The RI declined in patients with Graves' disease as hyperthyroidism was treated and TSH normalized, but never reached the values observed in healthy individuals. As in our study, Kurioka et al. observed no correlations between the flow parameters and serum FT3 and FT4 levels (19).

An increase in the peripheral resistance, expressed as an increase in RI, in both investigated patient groups suggests a direct influence of systemic hemodynamic disorders rather than that of local inflammation.

A study conducted by Nyirenda et al. has demonstrated that both Graves' disease patients and patients with toxic nodular goiter have a doubled risk of cardiovascular events. Additionally, toxic nodular goiter was also associated with increased risk of cerebrovascular disease (8). This risk was independent of the TSH concentration and hormonal compensation, and is probably associated with vascular endothelium injury due to chronic action of von Willebrant proinflammatory cytokines, thrombomodulin (19), and distorted sympathetic–parasympathetic balance in hyperthyroid patients (10). Moreover, a direct influence of thyroid hormones on vascular remodeling has been confirmed (24).

The results of PSV, EDV, and RI measurements in the OA in the Graves' disease patients were compared with the flow parameters in the patients with toxic nodular goiter and controls. A statistically significant increase in PSV and EDV was observed in the hyperthyroid patients, irrespective of the cause of hyperthyroidism, compared with the healthy individuals (*p* < 0.01 and *p* = 0.09, respectively). There was no difference in the RI values between these groups. Similar results were obtained in separate comparisons of the flow parameters between the Graves' disease patients and healthy controls as well as the patients with toxic nodular goiter and healthy individuals. There were no statistically significant differences between the groups of patients with hyperthyroidism of different etiologies. The lack of differences in the flow parameters in the hyperthyroid patients in both the ophthalmic and CRA indicates the hyperthyroidism-induced systemic influence on retrobulbar vessels. The results are in line with the reports of Kurioka et al. The comparison of RI values between Graves' disease euthyroid patients and healthy individuals revealed a statistically significant increase in RI values in the former group (19).

The lack of changes in RI values in OA in the hyperthyroid patients compared with the healthy individuals probably indicates a proportional increase in PSV and EDV, making RI values stable. The diameter of OA partially compensates the hyperkinetic flow, which makes RI values stable.

According to the reports, treated euthyroid patients still present changes in the peripheral flow parameters, which has been described by Kurioka et al. (19) and Nyirenda et al. (8). Our research confirmed the lack of differences in PSV, EDV, and RI between the hyperthyroid patients (TSH <0.35 uIU/mL) and patients with normal TSH levels (0.35–4.0 uIU/mL).

To conclude, the comparison of the flow parameters in two patient groups helped evaluate the influence of hyperthyroidism, irrespective of its cause, on the retrobulbar orbital vessels. Alterations in the flow parameters in the Graves' disease patients without any signs of clinically overt orbitopathy result from hemodynamic changes due to excessive hormone production. Irrespective of its cause and background, hyperthyroidism induces persistent changes in the cardiovascular system, even in euthyroid patients.

These changes are observed in hyperthyroid patients with Graves' disease and toxic nodular goiter. The present study involves the analysis of alterations in the flow parameters in the CRA and OA in hyperthyroidism of different etiologies. The results confirm earlier reports about statistically significant differences in PSV, EDV, and RI in the OA and CRA between patients with Graves' disease and healthy individuals. In addition, we demonstrate that in patients with toxic nodular goiter analogous changes occur in the CRA and OA also. These outcomes support the systemic influence of hyperthyroidism on the orbital vessels. The observed alterations in the flow parameters in the retrobulbar vessels are not dependent on serum TSH concentrations, i.e., even euthyroid patients present different values than healthy individuals.

There is a longstanding need to improve the classification of patients with thyroid orbitopathy, which would directly improve results of treatment and reduce complications. Appropriate ophthalmological classification has so far been based on the CAS scale and requires supplementation with new tools. Doppler ultrasound could provide a simple, non-invasive, repeatable, cheap and easily available test. However, its use would require a better understanding of which flow parameter changes in the orbital vessels result from local changes, and which are characteristic of hyperthyroidism and unrelated to thyroid orbitopathy. In this respect, we hope our study represents a step toward the establishment of new standards to improve the assessment of patients with Graves' disease and their eligibility for appropriate treatment.

Hence, further investigations addressing alterations of the flow parameters in the retrobulbar orbital vessels in Graves' disease should be compared not only with healthy controls but also with analogous values obtained in patients with toxic nodular goiter. This will enable the elimination of the systemic influence of hyperthyroidism and assure more reliable measurements.

## Data Availability Statement

The raw data supporting the conclusions of this manuscript will be made available by the authors, without undue reservation, to any qualified researcher.

## Ethics Statement

The studies involving human participants were reviewed and approved by Bioethic Comitee of Medical University of Warsaw (no. AKBE/201/17). The patients/participants provided their written informed consent to participate in this study.

## Author Contributions

DW-S: main researcher (contributed and design the study, performed the ophthalmic examination, doppler flow examination, and created the database of patients). GK: mathematical analysis of vascular flow and endocrine support. MM: performed the statistical analysis. JW: diagnosis of endocrine disease. IS-S: coordination of the research and correction of the draft. All authors contributed to manuscript revision, red and approved the submitted version.

### Conflict of Interest

The authors declare that the research was conducted in the absence of any commercial or financial relationships that could be construed as a potential conflict of interest.
